# Neural Substrates of the Drift-Diffusion Model in Brain Disorders

**DOI:** 10.3389/fncom.2021.678232

**Published:** 2022-01-07

**Authors:** Ankur Gupta, Rohini Bansal, Hany Alashwal, Anil Safak Kacar, Fuat Balci, Ahmed A. Moustafa

**Affiliations:** ^1^CNRS UMR 5293, Institut des Maladies Neurodégénératives, Université de Bordeaux, Bordeaux, France; ^2^Department of Medical Neurobiology, The Hebrew University of Jerusalem, Jerusalem, Israel; ^3^College of Information Technology, United Arab Emirates University, Al-Ain, United Arab Emirates; ^4^Research Center for Translational Medicine (KUTTAM), Koç University, Istanbul, Turkey; ^5^Department of Biological Sciences, University of Manitoba, Winnipeg, MB, Canada; ^6^School of Psychology & Marcs Institute for Brain and Behaviour, Western Sydney University, Sydney, NSW, Australia; ^7^School of Psychology, Faculty of Society and Design, Bond University, Robina, QLD, Australia; ^8^Faculty of Health Sciences, Department of Human Anatomy and Physiology, University of Johannesburg, Johannesburg, South Africa

**Keywords:** drift diffusion models, decision making, prefrontal cortex, basal ganglia, neural mechanism

## Abstract

Many studies on the drift-diffusion model (DDM) explain decision-making based on a unified analysis of both accuracy and response times. This review provides an in-depth account of the recent advances in DDM research which ground different DDM parameters on several brain areas, including the cortex and basal ganglia. Furthermore, we discuss the changes in DDM parameters due to structural and functional impairments in several clinical disorders, including Parkinson's disease, Attention Deficit Hyperactivity Disorder (ADHD), Autism Spectrum Disorders, Obsessive-Compulsive Disorder (OCD), and schizophrenia. This review thus uses DDM to provide a theoretical understanding of different brain disorders.

## Introduction

In daily life, we make numerous decisions; some are less complex, such as choosing a lane in the traffic. Others are very complex such as deciding which experiment to perform to investigate a research question best. Various studies attempt to understand decision-making on formal grounds using biophysical and abstract models. Biophysical models explore the interaction between the neural areas and the changes in the neurotransmitters such as GABA and glutamate (Jocham et al., 2012), while abstract models, such as the drift-diffusion model (DDM), attempt to explain the observed behavior (Ratcliff and Childers, 2015). One of these models, the DDM of decision-making, has gained significant attention. DDM estimates a number of model parameters such as decision threshold, drift rate, and bias based on the observed responses and the response time.

Several studies attribute DDM parameters to several cortical structures (Kim and Shadlen, 1999; Shadlen and Newsome, 2001; Kiani and Shadlen, 2009). These cortical areas work in conjunction with the basal ganglia (a subcortical structure) that provides auxiliary information to facilitate decisions and influence the DDM parameters. O'Connell et al. (2018) reviewed the link between neural activity in cortical and subcortical areas to DDM parameters. Computationally, Purcell and Palmeri (2017) show that based on neural activity, DDM parameters can be estimated, and vice versa. Additionally, Mulder et al. (2014a) reviewed non-invasive fMRI studies in humans to spotlight the role of various cortical and subcortical areas [prefrontal cortex, frontal eye field, striatum (STR), and pre-supplementary motor area (pre-SMA)] in controlling the DDM parameters. Another review showed that the posterior parietal cortex (PPC), frontal eye fields, dorsal STR, and lateral intraparietal area activity correlate to DDM parameters in humans and monkeys (Hanks and Summerfield, 2017). The authors further showed that in rodents, the rat PPC and frontal orienting fields (homologs of monkey PPC and frontal eye fields) could be neural correlates of DDM parameters (Hanks and Summerfield, 2017). These reviews corroborate the fact that these cortical and subcortical areas are involved in decision-making and are the sites for DDM parameters. A loss of structural and functional connectivity severely impairs the decisions in motor and cognitive tasks. One particular review focusing on imaging studies on psychosis provides a detailed account of the basal ganglia and the role of dopamine in explaining DDM parameters (Horga and Abi-Dargham, 2019). While there are many studies on DDM parameter changes in disorders, reviews on the topic are almost non-existent (Horga and Abi-Dargham, 2019). This review summarizes electrophysiological, behavioral, and imagining studies that correlate DDM parameters to the cortico-basal ganglia (BG) structures. Furthermore, we discuss DDM parameter changes in various disorders such as Parkinson's disease (PD), attention deficit hyperactivity disorder (ADHD), autism spectrum disorders (ASD), obsessive-compulsive disorder (OCD), and schizophrenia. Below, we first discuss the standard parameters of classical DDM briefly (Section The Drift-Diffusion Model). Following that, we discuss the neural substrates underlying each DDM parameter (Section Neural Substrates of DDM). Subsequently, we discuss how different brain disorders impact different DDM parameters (Section Brain Disorders).

## The Drift-Diffusion Model

**Figure 1:**
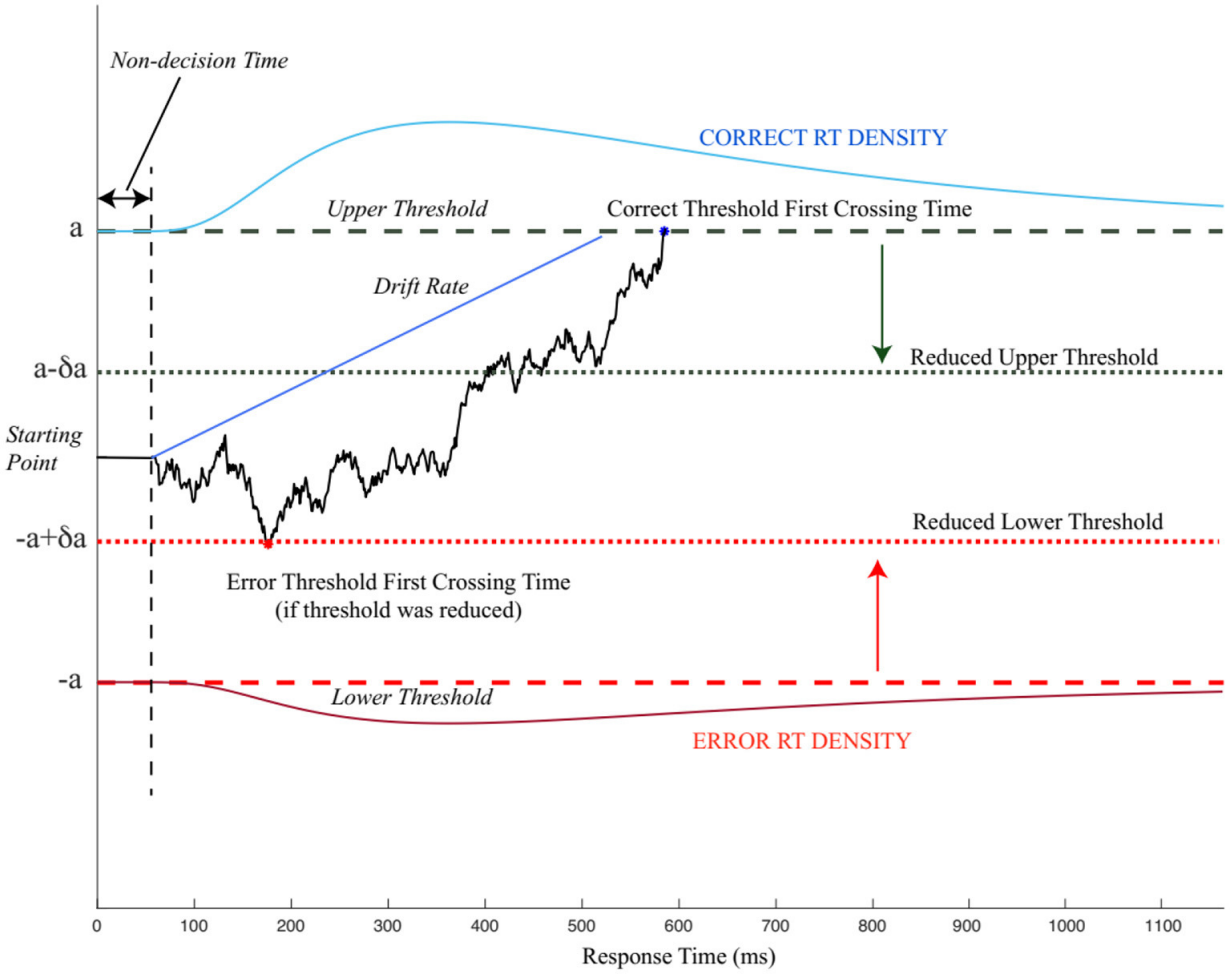
An illustration of the pure drift-diffusion model (DDM). The model accumulates evidence favoring two choices corresponding to correct choice (upper threshold) and incorrect choice (lower threshold). After the non-decision time, which is unrelated to the choices, the DDM starts accumulating evidence in favor of either choice (solid black line). When the evidence reaches either of the threshold (dashed black and red lines), the decision is terminated. The period between starting the evidence accumulation and threshold first crossing time refers to the decision time. If the threshold is reduced (dotted black and red lines), accumulating evidence may terminate the decision-making process earlier with incorrect choice selection modified from Wong et al. (2015).

The drift-diffusion model (also known as the diffusion decision model) was proposed by Ratcliff (1978) as an extension of early random walk models (e.g., Wald and Wolfowitz, 1948; Stone, 1960). The proposed model suggested that two-alternative forced-choice behavior can be modeled as a DDM, accumulating noisy evidence in favor of one alternative over another (Figure 1). The sequential addition of evidence enables the model to reach either of the choice-associated thresholds, terminate the evidence accumulation process, and select the choice (for which the threshold is crossed) as the preferred outcome (Ratcliff et al., 2016). The initial bias (or starting point) for evidence accumulation can influence the choice selection by moving the starting point closer to a decision threshold/choice, thereby making it farther away from the other threshold/choice. Bias toward a choice requires lesser evidence accumulation for the choice closer to the bias while requiring more evidence for the alternative choice before reaching the threshold. The drift rate or the rate of evidence accumulation determines how quickly the evidence reaches the decision threshold. Thus, the three decision-related parameters obtained from DDM are decision threshold, bias, and drift rate.

As with any computational model, the DDM also has several limitations, as discussed in Ratcliff et al. (2016). These limitations led to the development of many variants and alternatives of DDM (Usher and McClelland, 2001; Palmer et al., 2005; Wagenmakers et al., 2007; Ratcliff and McKoon, 2008; Vandekerckhove and Tuerlinckx, 2008; Wiecki et al., 2013). In this review, we focus on only the classical (standard) DDM model. The core parameters of the conventional DDM, including decision threshold, drift rate, bias, are discussed below.

### Decision Threshold

The decision threshold limits the amount of evidence gathered before decision finalization. While evaluating a stimulus to two decision threshold hypotheses, the decision-making system gathers evidence in favor of one hypothesis over the other in light of the information received. The decision terminates when one of the two decision thresholds, each representing a different hypothesis, is reached. Further empirical evidence also shows that these thresholds are dependent on multiple factors such as instructions emphasizing speed *vs*. accuracy (Mulder et al., 2013), task familiarity (Balci et al., 2011), and choice certainty/confidence (Philiastides et al., 2014; Lim et al., 2017).

One of the central concepts of decision-making research is the speed-accuracy tradeoff **(**SAT) paradigm, which shows that faster decisions are less accurate while slower decisions are more accurate. In a limited time-bound two alternate-forced-choice task, where time is of critical importance, participants must optimize the speed and accuracy to maximize rewards through training (Wald and Wolfowitz, 1948; Bogacz et al., 2006; Balci et al., 2011; Drugowitsch et al., 2012; Spieser et al., 2017; Desender et al., 2019; Riesel et al., 2019). The SAT is modulated within the framework of DDM by decision threshold setting (Mulder et al., 2014a). Specifically, reducing the threshold reduces the evidence required at the expense of task accuracy and *vice versa*. Earlier work shows that human participants can learn to optimize their decision by adopting the reward-rate maximizing decision threshold (Balci et al., 2011; Desender et al., 2019; Stafford et al., 2020).

In most time-constrained experiments, if a trial is not completed within a predefined time, the trial is considered a failure, and no rewards are obtained. In these time-constrained conditions, movements are often executed before the decision threshold is reached. The decision-making system accumulates evidence to refine or modify the motor output even after motor execution (Resulaj et al., 2009; Wong et al., 2015). The pre-decision movement execution enables a reduction of movement time while allowing for more online movement corrections.

The motor system prepares and executes the movement when certain choice confidence is reached to maximize movement execution and movement correction time. Even after movement execution, the model accumulates evidence to revalidate the selected choice (Krajbich and Rangel, 2011). Though many of the studies suggest that the decision threshold is fixed/constant, a few recent studies show that the threshold may not be fixed (O'Connell et al., 2018). One study indicated that during task execution, the thresholds are dynamically determined (Philiastides et al., 2014; Lim et al., 2017).

### Drift Rate

Evidence accumulation is a noisy process. A random Gaussian noise (with known mean and SD) is added at each step within a trial during the evidence sampling process. The drift rate is computed by averaging the rate of evidence accumulation from the start of evidence accumulation to the decision threshold. Averaging also serves the essential purpose of averaging out the instantaneous noise added. Therefore, the DDM parameter, drift rate, account for how quickly evidence is accumulated toward the correct decision threshold. By controlling for other parameters, higher drift rates mean shorter reaction times and higher accuracy. While choosing between rewarded and neutral stimuli (low conflict decisions), the drift rate was observed to be higher, and consequently, RT was found to be shorter (Ratcliff and Frank, 2012; Wiecki et al., 2013; Bond et al., 2018). Additionally, choice-value influences the drift rate, with high payoff decisions having higher drift rates (Starns et al., 2012; Bottemanne and Dreher, 2019). Drift rates also depend on the reliability of sensory evidence, with low-reliability evidence showing lower rates (Balci et al., 2011; Hanks et al., 2011; Drugowitsch et al., 2012; Mulder et al., 2012). In their study, Clay et al. (2017) showed that in high loss aversion (defined as increased sensitivity to losses), participants have a lower drift rate due to over fixation (which is related to higher evidence accumulation) on the non-selected choice even for easy trials (Clay et al., 2017). Additionally, with additional training on a task due to an increase in drift rate, a shorter RT is observed (Dutilh et al., 2009; Balci et al., 2011; Gur et al., 2020). During the aging process in rodents and humans, and/or lower accuracy is observed (Salthouse, 1996; Ratcliff et al., 2007a; McGovern et al., 2018; Gur et al., 2020; Theisen et al., 2020).

Together, these studies show that the drift rates are affected by stimulus quality, stimulus value, training, and the separation between choices.

### Bias

The starting point of evidence accumulation, or bias, plays a significant role in determining RT. A change in bias toward a favored choice (mathematically equivalent to reducing the threshold for the preferred choice) results in a lesser evidence requirement for reaching the threshold for favored choice and *vice versa*, thereby leading to faster RT and quicker decisions. Additionally, the task difficulty of the trial modulates the bias by favoring a more familiar choice (Mulder et al., 2012). The prior probability of associated payoffs is unknown in an unfamiliar task, and no prior memory exists to estimate the trial properties (Bogacz et al., 2006; Mulder et al., 2012). On repeating the trials, the stimulus characteristics are obtained, stored, and recalled for later trials (Simen et al., 2009; Leite and Ratcliff, 2011). Reward associated with the stimulus also influences the bias with higher value stimulus biasing the starting point (Mulder et al., 2012, 2014b; Rao et al., 2012; Fan et al., 2018). While bias is considered a static value predetermined before starting evidence accumulation, Hanks et al. (2011) showed that bias is dynamically altered in an ongoing trial to initiate faster RT.

## Neural Substrates Of DDM

For a site to qualify as an evidence accumulation area, the neuronal populations in the candidate brain area should increase their neural activity after stimulus presentation. This activity increase continues till a decision is reached, following which the neural activity returns to baseline (Tremel and Wheeler, 2015; Yartsev et al., 2018).

In this section, we discuss several cortical and subcortical basal ganglia areas that may show characteristics of being correlated with DDM parameters.

### Cortical Areas

The prefrontal cortex (PFC), frontal eye fields (FEF), lateral intraparietal area (LIP), superior colliculus (SC), fusiform, occipital, and inferior frontal gyri are observed to be sites for evidence accumulation (Ratcliff et al., 2003, 2007b; Tremel and Wheeler, 2015; Peel et al., 2017; Reppert et al., 2018). This neural activity indicating evidence accumulation in downstream elements is not a surprising finding as it may only mean that the upstream elements are involved in the ongoing decision-making process (Selen et al., 2012). This review explicitly discusses PFC, FEF, LIP, and SC in greater detail (see Table 1 for the summary).

**Table 1:** Frontal and basal ganglia areas, their connectivity, and effect on drift-diffusion model (DDM) parameters.

**Area**	**Projections from**	**Projections to**	**Parameter controlled**	**References**
FEF	LIP, MT, PFC	SC, PFC, STN	RT	Purcell et al., 2012; Peel et al., 2017; Hauser et al., 2018
			EA	Kim and Shadlen, 1999; Murd et al., 2020
			Decision commitment	Ding and Gold, 2012
LIP	SC	SC, FEF	Stimulus identity	Shushruth et al., 2018
			EA	Shadlen and Newsome, 2001; Meister et al., 2013; Zhou and Freedman, 2019; Zoltowski et al., 2019
			Confidence	Kiani and Shadlen, 2009
			RT	Zhou and Freedman, 2019
SC	LIP	LIP	EA	Ratcliff et al., 2003, 2007b; Peel et al., 2017; Reppert et al., 2018
			Confidence	Ratcliff et al., 2003; Odegaard et al., 2018
PFC	Thal, MT, FEF	Thal, STN, MT, FEF	EA	Henri-Bhargava et al., 2012; Vaidya and Fellows, 2015
			Confidence	Bang and Fleming, 2018; Shapiro and Grafton, 2020
			Stimulus valuation	Bechara et al., 1999; Fellows, 2006; Lim et al., 2011; Vaidya and Fellows, 2015; Bault et al., 2019; van Holstein and Floresco, 2020
			Cost of effort assignment	Vaidya and Fellows, 2015; Harris and Lim, 2016
			Drift rate	Wittkuhn et al., 2018
			Decision threshold	Georgiev et al., 2016; Wittkuhn et al., 2018
Pre-SMA	Sensory inputs	STR, STN, Thal	Decision threshold	Forstmann et al., 2008, 2010; Tosun et al., 2017; Berkay et al., 2018
Thal	PFC, pre-SMA, GPi	PFC, pre-SMA	Drift rate	Turner et al., 2015
STN	PFC, FEF, primary motor cortex, pre-SMA, GPe	GPi, GPe, PFC	Decision threshold (early termination)	Frank, 2006; Frank et al., 2007; Cavanagh et al., 2011; Tosun et al., 2017
GP	STR, STN	STN, Thal	Decision threshold	Kohl et al., 2015
			Drift rate	Kohl et al., 2015
STR	Sensory inputs, pre-SMA, SNc (dopamine inputs)	GPi, GPe	Value assignment	Lim et al., 2011; Westbrook et al., 2019
			Bias	Mulder et al., 2012; Wang et al., 2018; Zhang et al., 2019
			EA	Yartsev et al., 2018; Zhang et al., 2019
			RT	Nakamura and Hikosaka, 2006a
SNc		STR	RT	Frank and O'Reilly, 2006
			Decision threshold	See PD
			Drift rate	
			Early terminations of decisions	

*FEF, frontal eye fields; LIP, lateral intraparietal area; SC, superior colliculus; PFC, prefrontal cortex; SMA, supplementary motor area; STN, Subthalamic nucleus; GP, globus pallidus, GPi, globus pallidus interna; GPe, globus pallidus externa; STR, Striatum; Thal, Thalamus; SNc, substantia nigra pars compacta; MT, Middle temporal area; RT, reaction time; EA, evidence accumulation*.

#### Frontal Eye Fields

Many DDM studies are conducted in non-human primates where monkeys indicate the choice using eye movements. The FEF's involvement in eye movement and cognitive tasks has been widely studied. FEF receives inputs from PFC, LIP, middle temporal area (MT) while it outputs to PFC, STN, and SC (Purcell et al., 2010). Saccades showed longer latency with slow velocity and higher errors upon inhibiting FEF using muscimol (a GABA-A receptor agonist; Dias and Segraves, 1999). FEF inhibition reduces SC activity and increases overall RT (Peel et al., 2017). Neural activity in FEF and principal sulcus encodes decisions and performs evidence accumulation, as evident from persistent activity between 200 and 300 ms after cue onset until the saccade onset. Neural activity is modulated during movement toward the response field for low conflict conditions, where the evidence was mostly in favor of one of the two choices (Kim and Shadlen, 1999). To test whether the FEF integrates choice-specific outcomes or categorizes the evidence into discrete actions, Murd et al. (2020) stimulated FEF using TMS. The authors found that the task performance was affected through stimulation only during the choice-specific integration phase and not during the categorization phase. This study provides additional support for the role of FEF in evidence accumulation (Ding and Gold, 2012; Murd et al., 2020).

#### Lateral Intraparietal Area (LIP)

The LIP neuronal activity corresponds to sensory processing, memory processing, saccade-related responses, direction selectivity, and choices information (Shushruth et al., 2018). During a random dot motion (RDM) decision, where the participant indicates the direction of a cloud of dots motion, LIP neurons' firing rates were higher for correct choices when the motion was toward the receptive field. Interestingly, this activity increase was both before and during the motion display, indicating that LIP neurons access stimulus history for the decision-making process (Rao et al., 2012). The LIP activity was in the intermediate range on selecting an opt-out option (disbursing small yet guaranteed reward), suggesting the presence of confidence encoding neurons (Kiani and Shadlen, 2009). As LIP neurons are involved in evidence accumulation, these neurons combine both sensory and value information for a decision. LIP neurons' activity showed variations in response to the strength of sensory input and the value of the target (both in and outside the receptive field; Shadlen and Newsome, 2001; Zhou and Freedman, 2019; Zoltowski et al., 2019). As contrary to other reports showing that individual neurons are involved in evidence accumulation, Meister et al. (2013) found that the population-level activity in the LIP is more representative of the evidence accumulation.

#### Superior Colliculus (SC)

As SC has reciprocal connections with LIP, LIP suppression diminishes SC activity (Peel et al., 2017). Similar to LIP, increasing the number of distractors decreased overall activity in SC (Zylberberg et al., 2012). In decision-making, context confidence can be correlated to the probability of correct decisions. The confidence is measured through either opt-out trials (as discussed earlier) or self-reporting. Population-level activity shows that decision confidence and decision accuracy covary and SC only encodes decision accuracy and not subjective accuracy (Odegaard et al., 2018). Several studies implicate SC to evidence accumulation (Ratcliff et al., 2003, 2007b; Peel et al., 2017; Reppert et al., 2018; Schall, 2019).

#### Prefrontal Cortex

The ventromedial prefrontal cortex (vmPFC) plays a crucial role in value processing and preparatory activity by predicting, assigning, and dynamically updating the value to choices (Kable and Glimcher, 2007; Tusche et al., 2010; Henri-Bhargava et al., 2012; Selen et al., 2012; Bault et al., 2019; Shapiro and Grafton, 2020). The vmPFC also serves as an evidence accumulator and tracks the decision confidence (Henri-Bhargava et al., 2012; Vaidya and Fellows, 2015; Bang and Fleming, 2018; Shapiro and Grafton, 2020). Damage to the vmPFC impairs the value assignment to choices while comparing the choices remains unaffected (Fellows, 2006; Lim et al., 2011; Vaidya and Fellows, 2015; Bault et al., 2019). A separate study also found that vmPFC inactivation selectively increased risky choices for poorly rewarded outcomes (Bechara et al., 1999; van Holstein and Floresco, 2020).

The dorsomedial prefrontal cortex (dmPFC) assigns values and effort cost to unattended options for determining better options during exploration (Vaidya and Fellows, 2015; Harris and Lim, 2016; Bault et al., 2019). Additionally, the dlPFC (dorsolateral prefrontal cortex) is involved in evidence accumulation and associated with drift rate. Deactivation of dlPFC neurons using rTMS resulted in lower drift rates and impaired evidence accumulation (Wittkuhn et al., 2018). Continuous theta-burst stimulation (cTBS) of dlPFC (with inhibitory effect) decreased the drift rate only in high coherence trials (Georgiev et al., 2016). All these studies suggest the diverse roles of PFC neurons in processing different value-related parameters and accumulating evidence.

#### Pre-Supplementary Motor Area (preSMA)

Premotor areas are associated with the decision threshold modulation. For instance, Georgiev et al. (2016) found that cTBS of preSMA reduced the thresholds on trials, emphasizing accuracy. However, this was a counter-intuitive finding under the cortico-striatal theory of threshold modulation (for review, see Bogacz et al., 2010) since this manipulation would be expected to increase the threshold by reducing the excitability of the dorsal STR. The cortico-striatal theory of threshold modulation relies on the idea of the activation of the dorsal STR by the consistent cortical input coding for a particular action. The activation of the STR results in the disinhibition of globus pallidus interna (GPi), which at its resting state inhibits the thalamus and thereby cortex. The disinhibition of GPi results in the action execution by releasing the cortical areas associated with the desired action from inhibition while the other actions continue being inhibited (Forstmann et al., 2008). This functional architecture implements threshold modulation by modulating the likelihood of the desired action plan to be gated by basal ganglia. Thus, the inhibition of preSMA by cTBS would be expected to increase the threshold by reducing the excitability of the dorsal STR (Georgiev et al., 2016). Consistent with the cortico-striatal theory of threshold modulation, Tosun et al. (2017) and Berkay et al. (2018) found that the inhibition of preSMA using cTBS resulted in heightened decision thresholds in tasks where participants were asked to be as fast and as accurate as possible. Berkay et al. (2018) also found that increasing the excitability of the same brain region (by intermittent theta-burst stimulation) resulted in reduced decision thresholds. These findings were consistent with the correlational evidence that showed a relationship between pre-SMA and striatal activity and decision thresholds (Forstmann et al., 2008) and the relationship between pre-SMA-striatal connectivity and decision threshold modulation (Forstmann et al., 2008, 2010). Importantly, these effects were not observed with tDCS (Transcranial Direct-Current Stimulation) (de Hollander et al., 2016), which suggests a weaker efficacy of tDCS in modulating cortical excitability.

### Thalamus

We found only one study that correlated thalamic neural activity to DDM parameters. In a two-alternative forced-choice RDM (Turner et al., 2015), recorded fMRI activations and thalamus neural activity corresponded to the drift rate. BOLD activations were higher for the high drift rate trials compared to low drift rate trials. Additionally, this study found that thalamus activity was not indicative of the bias change (Turner et al., 2015).

### The Basal Ganglia

**Figure 2:**
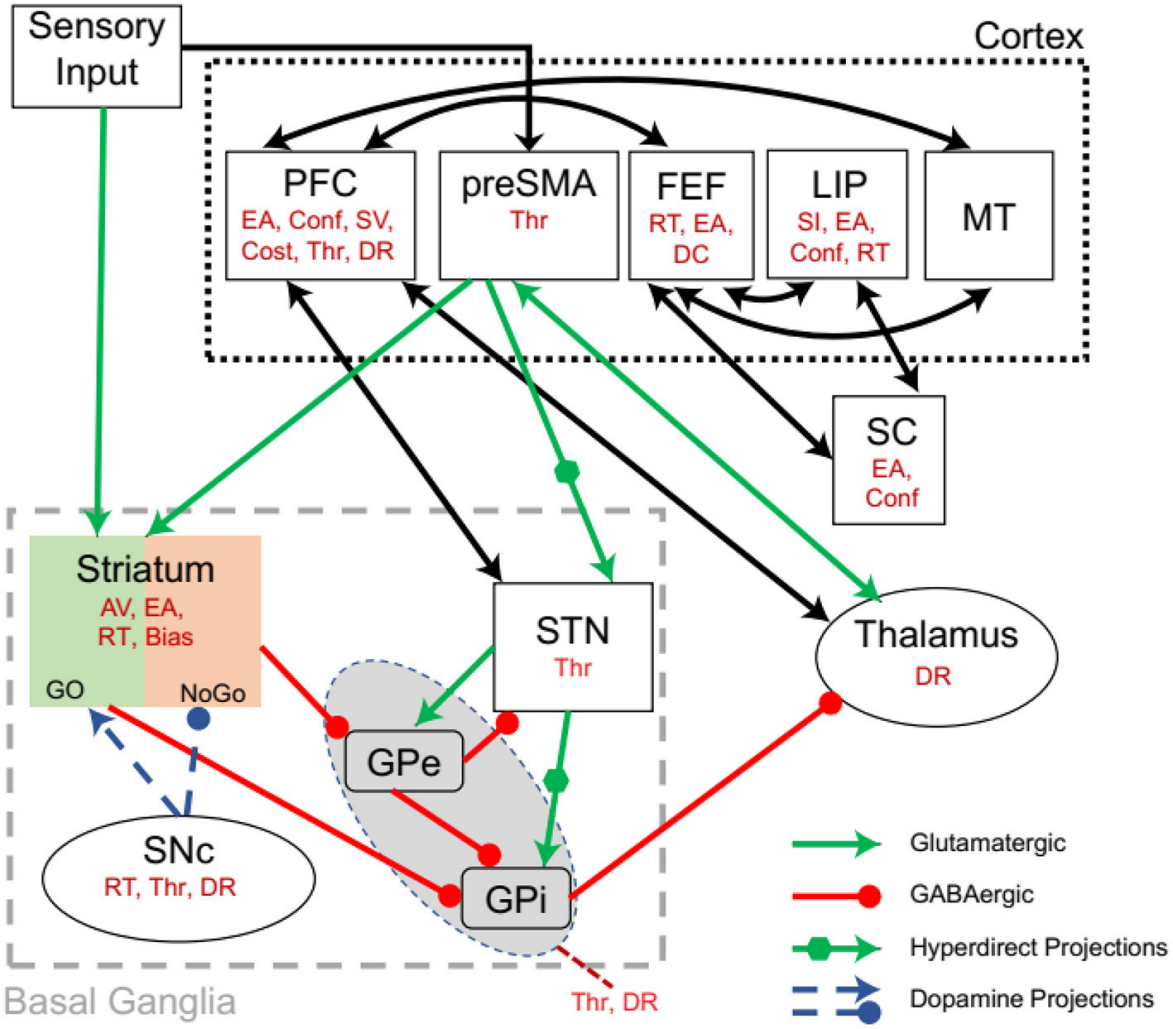
Interactions between the cortico-basal ganglia system showing the effect of an area on DDM parameters (in red). Based on the sensory inputs, the pre-supplementary motor area (pre-SMA) determines competing motor commands. Together sensory and pre-SMA inputs are projected to the striatum (STR). The pre-SMA also projects to subthalamic nucleus (STN) *via* a hyperdirect pathway. The action of dopamine from SNc modulates the Go and No-Go neurons in the STR. STR inhibits globus pallidus externa (GPe), which in turn inhibits globus pallidus interna (GPi). STR also inhibits GPi and STN has hyperdirect projections to GPi. GPi inhibits the thalamus. The STR-GPi pathways have an overall disinhibiting effect on the thalamus, while the STR-GPe-GPi has an overall inhibitory effect on the thalamus. PFC, Prefrontal Cortex; preSMA, pre-supplementary motor area; FEF, Frontal eye field; LIP, Lateral intraparietal area; MT, Middle temporal area; EA, Evidence accumulation; Conf, Confidence; SV, Stimulus valuation; Thr, Threshold; DR, Drift rate; AV, Action valuation; RT, Reaction time; selection; DC, Decision commitment; SI, Stimulus identity. Modified from Ratcliff and Frank (2012).

Recent studies highlight the role of the BG on DDM parameters. The BG (Figure 2) has several subregions, including STR, subthalamic nucleus (STN), globus pallidus (interna and externa), and substantia nigra (pars reticulata and pars compacta). These nuclei interact *via* excitatory and inhibitory connectivity, which result in a complex decision parameter control [for review, see (Moustafa et al., 2016a,b)]. The relation of BG on DDM parameters is presented below (for summary, also see Table 1).

#### Subthalamic Nucleus

The subthalamic nucleus receives inputs from PFC, FEF, primary motor cortex, and pre-SMA while it projects to ventral pallidum and GPi; STN also has bidirectional connections with the GPe (Benarroch, 2008). This connectivity pattern enables the functional coupling of these areas and the execution of sophisticated decision-making features. As the PFC and BG are distinct areas, the interaction between them is primarily controlled through the STN, which delays the BG gating to enable PFC to make the correct decisions and non-impulsive decisions (Frank, 2006; Frank et al., 2007). Additionally, during high conflict decisions, STN and PFC are functionally coupled (Zavala et al., 2013, 2014). The STN and vmPFC bias the decision toward a more valuable choice (Mulder et al., 2014b), and stimulation of STN lowers the decision threshold and terminates the decisions prematurely (Cavanagh et al., 2011).

#### Globus Pallidus

A study compared the DDM parameters changes in healthy controls, PD with GPi-DBS (Deep Brain Stimulation) ON and PD with GPi-DBS OFF. The authors suggested that the GPi controls decision threshold and drift rate (Kohl et al., 2015). As the decision threshold and drift rate changes were compared between the three groups mentioned above, the exact DDM parameter changes in healthy controls remain unknown. This is further discussed in Section Parkinson's Disease.

#### The Striatum

The STR is the primary input of the BG. A suppression of the caudate nucleus using muscimol selectively impaired the learning of new sequential motor procedures, while a suppression of the putamen selectively impaired learned motor sequence execution (Miyachi et al., 1997). Selectively inhibiting the D1 receptors in caudate increased saccadic RT for high reward expectation, and D2 receptor inhibition increased RT for small rewards (Nakamura and Hikosaka, 2006a). Interestingly, if stimulation followed the correct response, learning was enhanced (Nakamura and Hikosaka, 2006b). Optogenetic stimulation of the STR in mice performing a visual change detection task resulted in task performance changes by positively biasing the expected or valued visual events (Wang et al., 2018; Zhang et al., 2019). This bias is determined by the interactions between frontoparietal and frontostriatal circuits (Mulder et al., 2012). Another study involving behavioral, pharmacological, optogenetic, electrophysiological, and computational methods explored the role of the anterior dorsal STR in rodent auditory evidence accumulation tasks. The authors found that the anterior dorsal STR directly influences accumulation-based decisions with its neurons encoding for accumulated evidence throughout the decision process (Yartsev et al., 2018; Zhang et al., 2019).

#### The Substantia Nigra Pars Compacta

The substantia nigra pars compacta (SNc) is a critical area that is involved in dopamine production. SNc projections to STR enable dynamic modulation of DA-based switching between Go and No-Go pathways of the BG during cognitive learning (Frank and O'Reilly, 2006). D2 agonists enhanced the executive performance in low working memory patients while in high working memory participants, this effect was reversed (Gibbs and D'Esposito, 2005). Notably, the D_2_ antagonist increased Go learning while the opposite effect was seen for the D_2_ agonist. The effect of D_2_ agonists on D_2_ post-synaptic receptors results in the overall inhibition of the No-Go pathway leading to more Go selections and hence, faster RTs (Frank and O'Reilly, 2006). Additional indirect evidence for the SNc involvement comes from the studies on PD with medications. These studies show that the decision threshold, drift rate, early terminations of decisions are impacted by PD medications and are further discussed in Parkinson's Disease.

## Brain Disorders

In this section, we discuss how several brain disorders, including Parkinson's disease (PD), ADHD, ASD, OCD, and schizophrenia, and their associated neural damage impact DDM parameters. These diseases were selected as they impact the BG and PFC regions, and prior DDM studies on these patient population groups exist (see Table 2 for the summary).

**Table 2:** Drift-diffusion model parameters in different brain disorders.

**Disorder**	**Observed findings^‡^**
PD	↓ bias changes, ↓ drift rate, ↑decision threshold, ↓ non-decision time
PD on medication	↑ decision thresholdand ↓ drift rates, Early termination of decisions (impulsivity), ↔ RT, ↓ premotor + motor timings
PD off medication	↓ RT
PD on STN-DBS	↓ premotor + motor timings, Early termination of evidence during STN-DBS
PD on GPi-DBS	↑RT, ↓ drift rate, ↑threshold, ↔ in conflict resolution
Schizophrenia	↓RT, ↓drift rate during punishment trials, ↔ bias, ↔ decision threshold, ↓ stimulus history utilization, ↑non-decision time, ↑ Working memory use
ASD	↔ RT, ↔ drift rate, ↔ accuracy, ↑ decision threshold, ↓ post-error RT
OCD	↑ decision threshold, ↑ drift rate with signal strength, ↓ drift rate in high signal-to-noise coherence, and ↓ threshold after a penalty compared to controls. ↓drift rate which ↑ with coherence. ↓drift rate in children. ↑ RT
ADHD	↓ drift rate which is ↔ after stimulant medication ↓ decision threshold in accuracy trials, ↑ decision threshold in speed trials. ↑ RT in accuracy trials, ↔ RT in speed trials.

↑ *increased;*

↓ *decreased;*

↔ *no change*.

### Parkinson's Disease

Parkinson's disease (PD) is a BG motor disorder characterized by the loss of dopaminergic cells in the SNc. Though the primary impairment in PD is motor dysfunction (Moustafa et al., 2016a), several studies also show cognitive impairments in the disease (Moustafa et al., 2016b). When making decisions, utilizing the prior history of the stimulus to change the bias adequately is essential. Patients with PD are unable to use the previous information to make bias changes (Herz et al., 2016; Perugini et al., 2016). The patients with PD with hallucinations show lower drift rates, higher decision threshold, shorter non-decision time, along with inefficient and inflexible context-dependent evidence accumulation modulation (O'Callaghan et al., 2017). Levodopa medication shortens the premotor time and the motor time, enabling patients with PD to make faster yet inaccurate decisions (Rihet et al., 2002; Hasbroucq et al., 2003).

Huang et al. (2015) studied DDM parameter changes, particularly the drift rate and evidence accumulation in SAT, in medicated and non-medicated patients with PD. The authors presented participants with two versions of the moving-dots task for SAT and task difficulty. By changing the coherence between the moving dots, the task difficulty was altered. RT in all participant groups (healthy control and patients with PD) also increased when the task difficulty was increased. Although patients with PD had more performance errors, patients with PD on medication had more errors and lower drift rates than patients with PD off medication. This suggests that dopamine influences sensory information processing. In the SAT, PD patients off medication showed more errors in speed trials than accuracy trials and had slower RTs when compared to controls. No difference in the RT and errors was observed for patients with PD on medication and controls. A comparison of the DDM parameters revealed that both medicated and unmedicated patients with PD had lower drift rates and higher decision thresholds (Huang et al., 2015).

As discussed above (in section Subthalamic nucleus), STN delays BG gating until PFC makes the correct decision, and STN-DBS forces the decision to terminate early. Similar findings are also reported in patients with PD where STN-DBS and medication led to faster yet inaccurate decisions (Fluchère et al., 2018). RT fractionation showed that DBS reduced both premotor (stimulus onset to EMG onset) and motor time (EMG onset to movement onset; Fluchère et al., 2018). Herz et al. (2018) performed continuous and closed-loop DBS to STN. Patients with PD could adjust the decision threshold for difficult (high conflict) trials only when DBS was performed 400–500 ms after cue onset. Stimulation during this time window also enabled participants to make trial-to-trial adjustments and faster RT. However, for stimulation after 500 ms, the response time remained unaffected (Herz et al., 2018).

Globus pallidus interna -DBS significantly increased RT during response initiation, but the stop signal RT remained unchanged. Compared with controls, the drift rate was lower in both ON and OFF GPi-DBS groups. DBS stimulation also reduced the decision threshold (Kohl et al., 2015). For stop trials utilizing the No-Go pathway, the GPi-DBS-ON condition had a lower response delay. Still, DBS OFF was comparable to that of controls, and GPi-DBS did not affect the speed of conflict resolution (Kohl et al., 2015).

Patients with PD with hallucinations show a lower drift rate, increased decision threshold, and inflexible evidence accumulation modulation (O'Callaghan et al., 2017). Medications increase the speed but at the cost of accuracy. For speed favoring trials, the non-medicated patients with PD show a lower RT. Furthermore, medicated patients show decreased drift rate when compared to the non-medicated patients with PD. In both the medicated and non-medicated patients, the decision threshold is higher compared to controls. Like medication, STN-DBS resulted in faster and inaccurate decisions. PD participants could change the decision threshold when the stimulation was performed within a specific time range. GPi-DBS selectively increased RT for response initiation but not for response termination and reduced the decision threshold. At the same time, the GPi-DBS (both on and off groups) showed a reduced drift rate.

### Schizophrenia

Schizophrenia is characterized by altered sensory perceptions, cognitive impairments, and emotional dysregulation (Moustafa et al., 2016c, 2017; Ganguly et al., 2018). These symptoms originated from the dysfunction in the basal ganglia, frontal lobes, and temporal lobes (Buchsbaum, 1990). Post mortem studies in humans unveil increased striatal and globus pallidus volume in basal ganglia in paranoid hallucinatory schizophrenics. At the same time, no such changes were observed in the cortex and white matter (Heckers et al., 1991; Mamah et al., 2007). The higher volumes of these areas result from abnormal maturation that reduces basal ganglia volume during adolescence. Neuroleptic medications differing in their D_2_ receptor affinity also affect the striatal (caudate and STR) and globus pallidus volume. Therapeutic interventions using typical neuroleptics increased the BG volume. Treatment with atypical neuroleptic (such as clozapine) reduced the BG volumes over 2 years of treatment (Corson et al., 1999).

The drift-diffusion model fits show that patients with schizophrenia favored the accuracy over the speed with impaired learning on negative feedback (Moustafa et al., 2015). Using the reward *vs*. punishment learning task (in which participants learn to maximize the reward and minimize the punishments in different trials), Moustafa et al. (2015) showed that patients with schizophrenia had a slower RT due to slower encoding and slower motor time. Furthermore, a slower drift rate in punishment trials may be attributed to favoring accuracy, whereby patients were more cautious toward punishments. The authors also demonstrated that the initial bias in patients with schizophrenia and controls was similar, but participants failed to modify the bias based on the prior history of the stimulus (Moustafa et al., 2015). Further elaborating on this study, Fish et al. (2018) showed that the drift rate was lower and non-decision time was higher for patients with schizophrenia and their unaffected siblings. No significant difference in the initial bias and the decision threshold was observed for controls, patients with schizophrenia, and siblings (Fish et al., 2018).

Hierarchical (Bayesian) drift-diffusion model fit for billiard-ball collision timing reporting task suggests that the bias and not the drift rate was the main reason for premature response and impaired response inhibition that led to increased temporal estimation error (Limongi et al., 2018). The tradeoff favoring speed over accuracy conflicts with Moustafa et al. (2015); this may be due to the stricter time constraints imposed by Limongi et al. (2018).

People with schizophrenia favor the accuracy over the speed with slower RT and slower motor time. Additionally, participants showed impaired bias modulation and a lower drift rate for the punishment trials while the decision threshold remained unaffected. Incidentally, stricter timings cause premature response initiation, which may be related to bias but not drift rate.

### Autism Spectrum Disorders (ASD)

In ASD, the communication and behavioral impairment symptoms appear early during development. Some of the cognitive deficits in ASD persistently selecting the same choices, sensory hypersensitivity, and impaired interactions with dynamic objects (Sinha et al., 2014). These deficits arise due to abnormal sensory processing, spanning the superior temporal sulcus, fusiform face area, inferior parietal lobe, amygdala (AMY), extra striate body area in the lateral occipitotemporal cortex, PFC, and even BG (Gilbert et al., 2008; McPartland et al., 2011; Prat et al., 2016; Subramanian et al., 2017). It was demonstrated using a small sample study (comprising of only 7 ASD and six controls) that ASD children had 67% more PFC neurons with 79% more neurons in dlPFC and 29% more mPFC neurons when compared to neurotypical controls (Courchesne et al., 2011).

In numerical cognition tasks that involved indicating if an arithmetic equation is valid (2 + 3 = 5) or invalid (2 + 3 = 6), a positive correlation between fMRI activation in DLPFC and dmPFC, and numerical abilities was observed in ASD. At the same time, the healthy controls showed a negative correlation. In this study, accuracy and RT remained unchanged in ASD and controls while the decision thresholds were significantly higher (Karalunas et al., 2018; Iuculano et al., 2020). Pirrone et al. (2017) found that ASD participants showed higher thresholds while accuracy and drift rate were unaffected. Perhaps ASD participants follow a cautious approach by prioritizing accuracy over speed (Pirrone et al., 2017, 2020; Powell et al., 2019). After an error is encountered, RT for subsequent trials was slower. Comparing the post-error RT in 8–12-year-old children with high-functioning autism with controls revealed that the post-error RT is not impaired in high-functioning autism. fMRI analysis revealed that anterior medial PFC showed higher activation for error trials while the controls showed decreased activation (Goldberg et al., 2011). Overall, in ASD, the decision thresholds are higher, and the drift rate and RT remain unchanged.

### Obsessive-Compulsive Disorder (OCD)

Obsessive-compulsive disorder is a neuropsychiatric condition characterized by obsessions, intrusive thoughts, and compulsions, which are mental or behavioral acts that are hard to abstain from performing. The symptom profile in patients with OCD has a very diverse range. Therefore, the dimensional approach suggests four different symptom dimensions for OCD, namely symmetry/ordering, hoarding, contamination/cleaning, and obsessions/checking (Mataix-Cols et al., 2005). The literature is limited for studies investigating the decision-making processes of patients with OCD using DDMs. There are two studies conducted with subclinical OCD populations and four studies with patients with OCD. The response times of patients with OCD were found to be longer than healthy controls (Banca et al., 2015; Erhan et al., 2017; Mandali et al., 2019; Marton et al., 2019). The studies were conducted with subclinical OCD populations using the RDM and self-report questionnaires to assess participants' OCD-like features. The positive correlation between the scores gathered by the self-report questionnaire assessing OCD-like features and threshold was reported by both the studies (Erhan and Balci, 2017). The interaction between questionnaire score and drift rate increase with increased stimulus strength was also reported (Hauser et al., 2017).

One study compared patients with OCD and healthy controls using different coherence levels using the random-dot motion tasks (RDM) (Banca et al., 2015). Compared to healthy controls, the patients with OCD had a higher threshold in low signal-to-noise ratio conditions (coherence levels of 0.025 and 0.005) and lower drift rate in high signal-to-noise ratio conditions (coherence levels of 0.45 and 0.7). By using a similar experimental paradigm, Banca et al. (2015) and Marton et al. (2019) reported that patients with OCD show a lesser increase in drift rates as coherence increases (Marton et al., 2019). This finding implies that patients with OCD cannot fully utilize the signal in the stimulus. This study also does not report any significant difference in threshold settings between patients with OCD and healthy controls (Marton et al., 2019). The only research on the pediatric OCD population that used the DDM reported lower drift rates in children with OCD (Erhan et al., 2017). For post-error responses, patients with OCD have higher thresholds than healthy controls. On the other hand, the more pronounced decrease in decision threshold of patients with OCD compared to healthy volunteers was reported in the speed favoring (slow responses were penalized) SAT trials (Banca et al., 2015).

To our knowledge, the only study which does not use the RDM task but uses DDM utilizes the sequential learning task (Mandali et al., 2019). In this task, participants learned to differentiate the stimulus pairs associated with different reward rates. By considering the discrepancies in reward rates between pairs and distance of reward rate from chance levels, the conditions were segregated into conflict and certainty conditions. Patients with OCD performed poorly only in high conflict and high uncertainty conditions. Patients with OCD compared to healthy controls showed a lower drift rate only when the trials are complex, and reward probability is uncertain. In this condition, healthy controls responded quickly and randomly, but patients with OCD required more effort to find the correct answer.

To summarize, patients with OCD show increased RT, lower drift rate in high signal-to-noise ratio conditions, and higher decision threshold for low signal-to-noise ratio conditions. Though patients with OCD can modulate the drift rate for stimulus strength and coherence increase, they are unable to modulate it like controls. In children, lower drift rates and higher decision thresholds for post-error trials are observed.

### Attention-Deficit Hyperactivity Disorder (ADHD)

Attention-deficit hyperactivity disorder is a neuropsychiatric condition characterized by the symptom domains of inattention, hyperactivity-impulsivity, or both (American Psychiatric Association, 2013). ADHD is no longer perceived as a childhood disease, as the symptoms persist into adulthood for many patients. Some meta-analyses estimated the prevalence of ADHD among children as 7.2% and among adults as 2.5% (Simon et al., 2009; Thomas et al., 2015). The current theories for a neurobiological understanding of ADHD have a common suggestion for core issues in ADHD as dopamine (DA) alterations (Ziegler et al., 2016). The dopamine deficiency in ADHD is also compatible with the efficacy of stimulant medication (Wilens, 2008). A meta-analysis for functional neuroimaging studies of patients with ADHD reported a hypoactivity in the frontal areas (dorsolateral prefrontal, inferior prefrontal, and orbitofrontal cortex), dorsal anterior midcingulate cortex, superior parietal regions, caudate nucleus, and thalamus (Dickstein et al., 2006). These changes persist into adulthood except for a tendency for the improvement of caudate nucleus pathology (Kasparek et al., 2015).

The majority of studies reviewed by Ziegler et al. (2016) show a reduced drift rate and lower threshold in ADHD (Ziegler et al., 2016). The application of stimulant medication eliminated the differences in drift rates between ADHD and typically developing individuals (Fosco et al., 2017). Patients with ADHD were tested with random-dot motion tasks under the accuracy and speed emphasis (Mulder et al., 2010). Compared to typically developing children, patients with ADHD were faster under the accuracy emphasis, but the response times of these groups were similar in speed trials. DDM analysis revealed that patients with ADHD had lower thresholds in accuracy sessions and higher thresholds in speed sessions. This finding indicates that patients with ADHD do not optimize the speed-accuracy tradeoff as efficiently as control participants. The lower level of flexibility in the threshold setting of patients with ADHD was also reported by another study using a different paradigm (Weigard and Huang-Pollock, 2014). Weigard et al. used an implicit contextual cueing task and found that the thresholds of patients with ADHD were unresponsive to contextual cues. The decision-making and attentional alterations of adults with ADHD diagnosis were summarized in a meta-analysis (Mowinckel et al., 2015). Studies using simple perceptual decisions, reinforcement learning, risky decision-making, temporal discounting, and continuous performance tasks were analyzed by Mowinckel et al. (2015). The most significant effect size was found to reduce the drift rate (−1.62).

Therefore, in ADHD, the drift rate and decision threshold are reduced. The drift rate improved after taking stimulant medication. When performing accuracy trials, the ADHD group, compared to controls, had faster RT, while in speed trials, the two groups showed similar RT. ADHD participants also show impaired threshold adjustments for contextual cue changes.

## Discussion

Decision-making involves a selection of an outcome (from available choices) to maximize the rewards. Attempts to study the decision-making led to the development of various theoretical models such as the DDM. The DDM estimates the decision threshold, drift rate, and bias toward a choice based on the observed choices and reaction time. The electrophysiological, imaging, and behavioral experiments trace the DDM parameter computations to several cortical areas such as the FEF, LIP, SC, PFC, and pre-SMA. Neural activity in basal ganglia does not correspond to the determination of these parameters. BG facilitates decisions by choice valuation, assigning a cost to choice, learning an association between the choice and action, assigning the value to actions, signaling reward, task initiation, task termination, goal information presentation, sensory evidence gain modulation, the urgency to commit to a decision, integrating reward history to choices, selecting responses, determining utility, and learning a new task. DDM parameters are thus influenced by the close interactions between the cortical and BG areas. Impairment of any of the structures or a change in functional connectivity can lead to aberrations in decisions and the DDM parameters.

In PD, the loss of dopaminergic neurons in the SNc impact the RT, drift rate, and threshold. However, as SNc is a part of the more extensive BG network, almost all the DDM parameters are impacted. The increased threshold and slower drift rate result in patients being unable to gather enough evidence to cross a decision threshold or reach sufficiently high choice confidence for any of the available choices. This results in the patients continuing to gather evidence and may result in classical motor deficits like freezing of gait (Moustafa et al., 2016a,b). It has been further observed that when the patients are presented with additional visual information like strips on the floor, they can overcome the freezing (Cao et al., 2020). The additional visual cues may alleviate the freezing by reducing the decision threshold. PD medications lower the threshold, which results in decisions terminating without enough evidence gathering. This is consistent with the various cognitive and motor studies suggesting the medication increases the impulsivity in PD. In Schizophrenics, a lower BG volume and reduced BG activation are observed. This results in reduced activation of the STR and reduced functional connectivity between the BG and PFC, which leads to abnormal action valuation (Bernard et al., 2017). The valuation change impairs the patient to integrate the prior trial information and modulate the bias.

As the pathology is arguably limited mainly to the STR, the decision threshold remains largely unaltered. Furthermore, the altered D2 affinity in Schizophrenics disinhibits the NoGo pathways (which is involved in punishment learning), and therefore, the Schizophrenics follow the punishment avoidant approach (Cox et al., 2015). Current neurobiological understanding of OCD implies the serotonergic dysfunction-related hyperactivity of cortico-striato-thalamocortical pathways (Dougherty et al., 2018). A reduction in serotonin level interrupts its inhibitory role in the STR, thalamus, and cortical areas, resulting in hyperactivation of cortico-striato-thalamocortical loops. The cortico-striato-thalamocortical loops involved in the pathophysiology of OCD are particularly indicated as the caudate nucleus, dorsal anterior cingulate cortex, and orbitofrontal cortex (Dougherty et al., 2018). These structures are found to be abnormally active at rest and with symptom provocation (Dougherty et al., 2018). One theory suggests that compulsions are a way to relieve anxiety caused by obsessions. The source of anxiety in patients with OCD is reported to be caused by amygdala hyperactivity (Simon et al., 2010). Another approach to explain OCD symptoms is based on the feeling of incompleteness to counteract “not-just right experiences.” Low-frequency rTMS application to pre-SMA reduced the incompleteness-driven symptoms (Mantovani et al., 2013). In ASD, the increase in PFC neurons (Courchesne et al., 2011) and reduced volumes of BG and thalamus (Estes et al., 2011) result in higher decision thresholds. In ADHD, hypoactive PFC, BG, and thalamus (Zhu et al., 2016) and the impaired functional connectivity between the STR and thalamus (Mills et al., 2012) results in lower decision threshold and drift rates.

Many of the motor and cognitive processes leverage the interaction between cortical and BG areas; structural and/or functional damage can lead to motor and cognitive deficits leading to many disorders. In this review, we highlight how the DDM parameters differ in various disorders. The studies featured in this review present a significant understanding of the neural basis of DDM, including cortical and BG areas. Given the close interactions between frontal cortical regions and BG, it is of utmost importance that both frontal and BG areas are studied together in future DDM studies.

Future studies should be directed toward probing multiple DDM parameters and several BG and PFC areas using microelectrode arrays and finding interactions between all the regions. As discussed above, simulation-based functional inactivation of each of these areas will further refine our understanding of various clinical disorders. DDM parameters provide a robust estimate of decision properties that can be used to estimate cognitive performance quantitatively. These quantitative estimates may enable tracing the latent variables, which allow for making mechanistic inferences about various therapies, disease progression, and efficacies of neurostimulation.

## Author Contributions

All authors listed have made a substantial, direct, and intellectual contribution to the work and approved it for publication.

## Funding

This work received financial support from the United Arab Emirates University (grant no. CIT 31T129).

## Conflict of Interest

The authors declare that the research was conducted in the absence of any commercial or financial relationships that could be construed as a potential conflict of interest.

## Publisher's Note

All claims expressed in this article are solely those of the authors and do not necessarily represent those of their affiliated organizations, or those of the publisher, the editors and the reviewers. Any product that may be evaluated in this article, or claim that may be made by its manufacturer, is not guaranteed or endorsed by the publisher.
